# Multipyrene Tandem Probes for Point Mutations Detection in DNA

**DOI:** 10.1155/2013/860457

**Published:** 2013-12-24

**Authors:** Svetlana A. Kholodar, Darya S. Novopashina, Mariya I. Meschaninova, Alya G. Venyaminova

**Affiliations:** Institute of Chemical Biology and Fundamental Medicine SB RAS, Lavrentiev Avenue 8, Novosibirsk 630090, Russia

## Abstract

Here we report design, synthesis and characterization of highly sensitive, specific and stable in biological systems fluorescent probes for point mutation detection in DNA. The tandems of 3′- and 5′-mono- and bis-pyrene conjugated oligo(2′-O-methylribonucleotides), protected by 3′-“inverted” thymidine, were constructed and their potential as new instruments for genetic diagnostics was studied. Novel probes have been shown to exhibit an ability to form stable duplexes with DNA target due to the stabilizing effect of multiple pyrene units at the junction. The relationship between fluorescent properties of developed probes, the number of pyrene residues at the tandem junction, and the location of point mutation has been studied. On the basis of the data obtained, we have chosen the probes possessing the highest fluorescence intensity along with the best mismatch discrimination and deletion and insertion detection ability. Application of developed probes for detection of polymorphism C677T in MTHFR gene has been demonstrated on model systems.

## 1. Introduction

The design of tools for point mutations (SNP, one nucleotide insertion, or deletion) detection is a problem of current importance. Recently, various techniques for genetic diagnostics have been developed [1–3]. A large part of these methods is based on hybridization of fluorescent oligonucleotide probe to its complementary DNA target to generate fluorescent signal. Pyrene conjugates of oligonucleotides as potential diagnostic probes attract attention of researchers due to the unique properties of pyrene such as long fluorescence lifetime, considerable sensitivity to the microenvironment, high quantum yield, and ability to form excimers and exciplexes [4].

Earlier, tandems of monopyrene-coupled oligonucleotides have been designed for nucleic acids probing [5–7] and SNP diagnostics [8–11]. The principle of action of these probes is based on the excimer formation at the tandem junction where a pyrene unit on the 3′-terminus of one component of the probe interacts with a pyrene unit on the 5′-terminus of another component. Tandem excimer probes are of particular interest in case of detection of such subtle structural alterations caused by point mutations in DNA-tandem complexes. In contrast to tandem probes relying on fluorescence resonance energy transfer (FRET) [12] which is realized within 1–10 nm, excimer tandem probes rely on formation of excimer between two parallel pyrene units at a distance of only 3.4 Å which almost corresponds to the length of the one base pair along the DNA strand.

**Figure 1:**
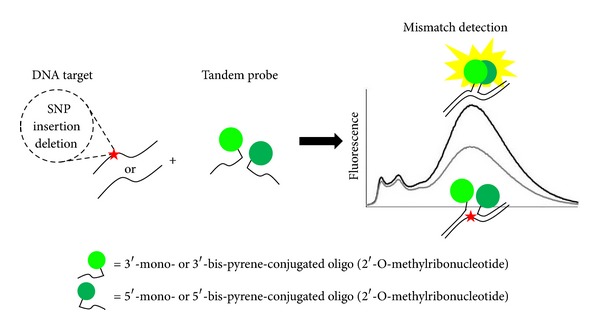
The scheme of the proposed concept of multipyrene tandem probes action.

We have been interested in design of the novel type of pyrene-coupled tandem as point-mutation probes possessing new useful qualities. Oligo(2′-O-methylribonucleotides) were chosen as a basis for these probes due to their numerous advantages including stability to endonucleases, high affinity to nucleic acids, and high rate of hybridization [13]. Besides, oligo(2′-O-methylribonucleotides) are remarkable for their increased ability to discriminate mismatches in comparison to oligodeoxyribonucleotides [14]. Additionally, we have modified the probes' 3′-termini by thymidine attached via a 3′-3′-phosphodiester internucleotide bond (3′-“inverted” thymidine) to achieve greater stability to exonucleases [15–17]. Previously we have reported the construction of tandems of two bis-pyrene conjugated oligo(2′-O-methylribonucleotides) as potential fluorescent SNP biosensors [18, 19]. This study is targeted to the application of tandem probes relying on excimers formation between 2 to 4 pyrene units at the tandem junction for detection of point mutations in DNA target by fluorescence emission and melting curve analysis with detection by excimer fluorescence change. We have studied the effect of variation of number of pyrene units at the tandem junction and the location of the junction on the abilities of proposed tandem probes to detect SNP, one nucleotide deletions and insertions (see Figure 1).

Methylenetetrahydrofolate reductase gene (MTHFR) C677T SNP was chosen as a subject of detection in our research. This polymorphism is widespread among the population of the Earth and is associated with inborn neural tube defects [20], cardiovascular diseases risk [21], pregnancy complications [22], and cancer [23, 24]. Thus, we have applied proposed fluorescent probes to detect the C677T polymorphism in a model system using 41-mer oligodeoxyribonucleotides corresponding to 651–691 MTHFR gene fragment. Additionally, we used oligodeoxyribonucleotide targets with single nucleotide insertions and deletions to estimate the potential of proposed probes to detect corresponding mutations.

## 2. Materials and Methods

### 2.1. Materials

Oligo(2′-O-methylribonucleotides) and oligodeoxyribonucleotides were synthesized on the ASM-800 synthesizer (Biosset, Novosibirsk, Russia) by the solid phase phosphoramidite method according to protocols optimized for this synthesizer with 3′-*β*-cyanoethyl-N,N-diisopropylphosphoramidites of 5′-O,N-protected 2′-O-methylribonucleosides, deoxyribonucleosides, and CPG-500 nucleoside-bound polymeric carriers (Glen Research, USA). Oligo(2′-O-methylribonucleotides) containing 3′-“inverted” thymidine were synthesized using 3′-O-dimethoxytritylthymidine-coupled CPG prepared by analogy with [25]. 3′-O-Dimethoxytritylthymidine was synthesized as in [26]. 3′-Phosphorylated oligo(2′-O-methylribonucleotides) III, IV were synthesized with [2-[2-(4,4′-dimethoxytrityloxy)ethyl]sulphonyl]ethanol-coupled CPG prepared by analogy with [25]. [2-[2-(4,4′-Dimethoxytrityloxy)ethyl]sulphonyl]ethanol and its H-phosphonate were synthesized according to [27, 28]. 5′-Phosphorylation of oligo(2′-O-methylribonucleotides) I, II was performed by analogy with [27]. The molar extinction coefficients of pyrene conjugates were calculated as the sum of the molar extinction coefficients of the corresponding oligonucleotides and that of one or two pyrene residues attached to the oligomer (24000 M^−1^ cm^−1^ at 260 nm) [28, 29]. Synthesized conjugates were characterized by MALDI TOF MS spectra recorded with REFLEX III (Bruker Daltonics, Germany) mass spectrometer. Anthracene and 9,10-diphenylanthracene were obtained from Sigma-Aldrich (USA).

### 2.2. Synthesis of 5′- and 3′-Pyrenylmethylphosphoramide Derivatives of Oligo(2′-O-methylribonucleotides) Ia–IVa

N,N-Dimethylaminopyridine (DMAP) (5 mg, 41 *μ*mol) in anhydrous DMSO (50 *μ*L), triphenylphosphine (6.8 mg, 25 *μ*mol) in anhydrous DMSO (25 *μ*L), and 2,2′-dipyridyl disulfide (5.3 mg, 25 *μ*mol) in anhydrous DMSO (25 *μ*L) were added to a cetyltrimethylammonium salt of 3′- or 5′-phosphate of oligo(2′-O-methylribonucleotides) I–IV (2-3 OE_260_, approximately 0.03 *μ*mol). The reaction mixture was stirred for 15 min at 56°C. Oligo(2′-O-methylribonucleotide) with terminal activated phosphate was precipitated with 2% LiClO_4_ in acetone, quickly washed with acetone, and dissolved in water (5 *μ*L). 1-Pyrenylmethylamine hydrochloride (2 mg, 7.5 *μ*mol) in the mixture of anhydrous DMSO (20 *μ*L) and anhydrous TEA (5 *μ*L) was added to activated phosphate of oligo(2′-O-methylribonucleotide). The reaction mixture was stirred for 2 h at 56°C. Oligonucleotides were precipitated with ether and reprecipitated two times from 0.15 M aqueous solution of LiClO_4_ with 2% LiClO_4_ in acetone. The obtained derivatives were purified by preparative 20% denaturing PAGE with subsequent precipitation as lithium salts (see Table 1).

### 2.3. Synthesis of 5′- and 3′-Bispyrenylmethylphosphorodiamide Derivatives of Oligo(2′-O-methylribonucleotides) Ib–IVb

DMAP (5 mg, 41 *μ*mol) in anhydrous DMSO (50 *μ*L), triphenylphosphine (6.8 mg, 25 *μ*mol) in anhydrous DMSO (25 *μ*L), and 2,2′-dipyridyl disulfide (5.3 mg, 25 *μ*mol) in anhydrous DMSO (25 *μ*L) were added to a cetyltrimethylammonium salt of 3′- or 5′-phosphate of oligo(2′-O-methylribonucleotide) I–IV (2-3 OE_260_, approximately 0.03 *μ*mol). The reaction mixture was stirred for 15 min at 56°C. 1-Pyrenylmethylamine hydrochloride (2 mg, 7.5 *μ*mol) in the mixture of anhydrous DMSO (20 *μ*L) and anhydrous TEA (5 *μ*L) was added to oligo(2′-O-methylribonucleotide) with terminal activated phosphate. The reaction mixture was stirred for 2 h at 56°C. Oligonucleotides were precipitated with ether and reprecipitated two times from 0.15 M aqueous solution of LiClO_4_ with 2% LiClO_4_ in acetone. The obtained derivatives were isolated as described above (see Table 1).

### 2.4. Quantum Yield Determination

The fluorescence quantum yields were measured by the relative method using 9,10-diphenylanthracene (Φ_*F*_ = 1.0) in cyclohexane as the first standard and anthracene (Φ_*F*_ = 0.36) in cyclohexane as the second standard [30]. 9,10-Diphenylanthracene and anthracene were used without recrystallization. Solutions of standards in cyclohexane and conjugate samples in 6.7 mM Tris-HCl (pH 8.9), 16 mM (NH_4_)_2_SO_4_, 3.6 mM MgCl_2_ had not been degassed before quantum yield determination. The fluorescence quantum yields of 5′- and 3′-mono- and bis-pyrene-labeled oligo(2′-O-methylribonucleotides) Φ_*F*_(PyrN^*m*^) were determined according to following equation:

(1) ΦF(PyrNm)=ΦF(A)·A(PyrNm)A345(PyrNm)·1α(ANT)·n(H2O)2n(cyclohexane)2.

Φ_*F*_(*A*) designates a cross-calibrated value for the fluorescence quantum yield of anthracene in cyclohexane; *A*(PyrN^*m*^) stands for the fluorescence emission spectra of the sample from 360 to 600 nm; *A*_345_(PyrN^*m*^) is the absorbance of the sample at the excitation wavelength of 345 nm; *α*(ANT) is a slope of the fluorescence emission area versus *A*_345_ calibration curve of anthracene; *n*(H_2_O) is a refractive index of water, *n*(H_2_O) = 1.3328; *n*(cyclohexane) is a refractive index of cyclohexane, *n*(cyclohexane) = 1.4266.

### 2.5. UV-Absorbance Measurements

UV-spectra were recorded with 2 · 10^−6^ M solutions in 6.7 mM Tris-HCl (pH 8.9), 16 mM (NH_4_)_2_SO_4_, and 3.6 mM MgCl_2_ in quartz cuvettes with the optical length of 10 mm at room temperature on Shimadzu UV-2100 Spectrophotometer (Kyoto, Japan).

### 2.6. Thermal Stability Measurements

Thermal stability of duplexes was studied with 1 · 10^−6^ M solutions of probe and DNA target in 6.7 mM Tris-HCl (pH 8.9), 16 mM (NH_4_)_2_SO_4_, and 3.6 mM MgCl_2_ on Cary 300 BioMelt (Varian, Inc., Australia). The rate of temperature change was 0.5°C/min. The temperature in the cuvettes was determined with thermocouple Temperature Probes Series II (Varian Inc., Australia). The melting temperature of oligonucleotide complex corresponded to the maximum of the first derivative of the melting curve of this complex obtained by measuring absorbance at 260 nm against increasing temperature. Absolute error of *T*_*m*_ determination did not exceed 0.3°C.

### 2.7. Fluorescence Measurements

Fluorescence spectra and melting curves were recorded with 2 · 10^−7^ M solutions in 6.7 mM Tris-HCl (pH 8.9), 16 mM (NH_4_)_2_SO_4_, and 3.6 mM MgCl_2_ in quartz cuvettes with the optical path length of 4 mm at a voltage of 600 V on Cary Eclipse Fluorescence Spectrophotometer (Varian Inc., Australia) and thermostating with Thermostatic circulator 2219 Multitemp II (LKB Bromma, Sweden). The probes were preliminary annealed at 95°C and cooled for 45 min to room temperature. The emission spectra were recorded in the range of 360 to 600 nm at an excitation wavelength of 345 nm. The fluorescence values were registered at 480 nm at the same excitation wavelength on registration of fluorescence melting curves. The thermal dependences were obtained on heating of samples at a rate of 0.7–1.5°C/min. Melting curves were processed with OriginPro 7.5 (OriginLab, USA). Relative error of fluorescence intensity determination did not exceed 5%.

## 3. Results and Discussion

### 3.1. Design of Tandem Fluorescent Probes

The 5′- and 3′-mono- and bis-pyrene-labeled oligo(2′-O-methylribonucleotides) were proposed as components of the new tandem fluorescent probes. Sequences of oligomers were chosen to be complementary to a fragment within 651–691 region of MTHFR gene. The terminal phosphate of oligo(2′-O-methylribonucleotides) has been chosen as a position of labeling as such coupling does not prevent conjugates from forming stable duplexes with DNA targets. We have selected a convenient postsynthetic method of terminal phosphate activation by oxidation-reduction pair triphenylphosphine-dipyridyldisulfide in the presence of nucleophilic catalyst N,N-dimethylaminopyridine followed by the reaction with ligand bearing nucleophilic group [31].

The 5′- and 3′-phosphorylated oligo(2′-O-methylribonucleotides) were synthesized by phosphoroamidite automatic synthesis. 5′-Phosphorylated oligomers contained 3′-“inverted” thymidine to increase their stability towards 3′-exonucleases. Coupling of pyrene residues to 5′- or 3′-terminal phosphate of oligo(2′-O-methylribonucleotides) was realized by analogy with [29], using greater excess of 1-pyrenylmethylamine to increase the yields of the conjugates. In case of monopyrene-labeled conjugates Ia–IVa synthesis the activation of terminal phosphate was followed by removal of activating reagents and nucleophilic catalyst excess. The subsequent reaction was carried out in a water-organic medium. The activated intermediate derivative was dissolved in water and 1-pyrenylmethylamine in DMSO/TEA mixture was added. In case of bis-pyrene-labeled conjugates Ib–IVb synthesis the reaction between oligo(2′-O-methylribonucleotides) bearing activated terminal phosphate and 1-pyrenylmethylamine was performed in organic medium without removal of activating reagents and the catalyst.

The reaction mixtures in all cases were analyzed by PAGE. The 5′- and 3′-mono- and bis-pyrene-labeled oligo(2′-O-methylribonucleotides) were purified by preparative PAGE, desalted, and obtained as lithium salts. The structures of synthesized conjugates were confirmed by the combination of massspectrometry (see Table 1) and UV- and fluorescent (see Figure 3) spectroscopy. Quantum efficiencies (Φ_*F*_) of conjugates were determined relatively to 9,10-diphenylanthracene as a first standard and anthracene as a second standard (see Table 1).

**Figure 2:**
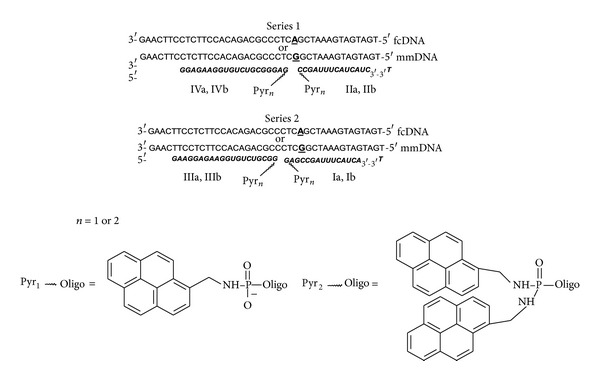
Duplexes of tandem probes and DNA targets. N-deoxyribonucleotide, ***N*** = 2′-O-methylribonucleotide. mmDNA = DNA target with mononucleotide substitution; fcDNA = fully complementary DNA target. IVa, IVb, IIIa, IIIb = 3′-components, IIa, IIb, Ia, Ib = 5′-components.

Tandem probes were constructed on the basis of synthesized pyrene conjugates (see Figure 2). Two series of tandem probes differing in the location of tandem junction (Series 1 and Series 2, Figure 2) were designed. In Series 1 mismatch position corresponded to the first nucleotide from the tandem junction. In Series 2 mismatch position was four nucleotides shifted from the tandem junction. Thus, the effect of the tandem junction location relatively to the mismatch position was studied. The model DNA targets containing the two variants of one nucleotide deletion (Del3 and Del 5) and four variants of insertions (InsA, InsG, InsT, and InsC) were synthesized to investigate the possibility of these point mutations detection using tandem probes of Series 2.

### 3.2. Thermal Stability of Duplexes of Tandem Probes with DNA Targets

The thermal stability of duplexes between fluorescent probes and DNA targets plays an important role in point mutations analysis. Upon the formation of duplex between pyrene conjugate of oligonucleotide and NA target, pyrene unit can either participate in stacking interactions with terminal base pairs of duplex [32, 33], intercalate into the duplex [34–39], or locate in one of the grooves [40, 41]. If pyrene residue is attached to the terminal nucleoside it is expected to be in stacking with the terminal base pairs of the duplex. Previously, we have shown that introduction of one or two pyrenylmethylamine residues into the 5′-terminal phosphates of oligo(2′-O-methylribonucleotides) leads to the increase of the thermal stability of their duplexes with DNA targets [29]. In case of triple complexes of tandem probes with DNA targets, terminal pyrene residues located at the junction can be seen as ones inserted into the nucleosides in the center of oligonucleotide chain. Generally, pyrene residues introduced into the nucleosides or nonnucleoside inserts inside the oligonucleotide chain intercalate into the duplex. This in turn results in the increase of the thermal stability of the duplex. The effect of introducing of two or more pyrene residues into the tandem junction on the thermal stability of tandem·NA duplexes has never been studied before. In this work the relationship between thermal stability of tandem duplexes with DNA targets and number of pyrene residues at the tandem junction has been investigated. As a test system we used tandems of Series 2 and their individual components. As controls devoid of pyrene residues we used 3′- and 5′-phosphorylated oligo(2′-O-methylribonucleotides) IV and II, correspondingly.

Tandems of oligonucleotides are known to form more thermally stable duplexes in comparison to single oligonucleotides due to the cooperative interactions between the components of tandems [42]. As consistent with this property of tandems, an increase of melting temperature (*T*_*m*_) for the studied tandem duplex (IV+II)·fcDNA was as significant as 11.3°C in comparison with *T*_*m*_ value for duplex of individual tandem component II with fcDNA (see Table 2). Here, the presence of both 5′- and 3′-phosphates at the tandem junction is expected to be a favorable factor in terms of cooperative interactions between tandem components, as phosphate groups shield the inner hydrophobic region of double helix from the unfavorable hydrophilic surrounding [43]. An introduction of one pyrene residue to the 5′-component IIa resulted in reduction of *T*_*m*_ value of corresponding duplex (IV+IIa)·fcDNA by 4.5°C, thus, allowing to assume a pyrene unit to disturb cooperative interactions of tandem components. On the contrary, good cooperative interaction was observed upon introduction a single pyrene residue to the 3′-component as follows from comparison of *T*_*m*_ values of (IVa+II)·fcDNA and (IV+II)·fcDNA duplexes. Thus, attachment of 1-pyrenylmethylamine residue to the 3′- or 5′-component of tandem has a nonequivalent and rather a destabilizing effect on the thermal stability of the resulting duplexes. It is necessary to indicate that in case of introduction of bis-pyrene group to one of the tandem components we found more prominent but different destabilizing effect in comparison to introduction monopyrene group. Bis-pyrene group in the 3′-component of tandem resulted in the significant reduction of stability of duplex (IVb+II)·fcDNA (Δ*T*_*m*_ 7.5°C in comparison with *T*_*m*_ of duplex of tandem devoid of pyrene residue). On the contrary, not much difference between thermal stabilities of duplexes (IV+IIb)·fcDNA and (IV+IIa)·fcDNA has been found. Considerably more complex cases arise if pyrene residues are attached to both of the tandem components. We observed *T*_*m*_ value for (IVa+IIa)·fcDNA duplex to be 3.6°C lower than *T*_*m*_ value of corresponding duplex devoid of pyrenes ((IV+II)·fcDNA). This may be explained by the previously found destabilizing effect of 5′-pyrene component. An insignificant increase of stability in comparison to (IV+IIa)·fcDNA may be due to stacking interactions between pyrene units at the junction. While stacking of pyrene units is obvious as it is also confirmed by the appearance of excimer fluorescent signal, it does not result in appreciable stabilization effect in this case. Duplexes of tandems with 3 pyrene units at the junction (IVb+IIa)·fcDNA and (IVa+IIb)·fcDNA were not substantially stabilized either, while a little tendency to stabilization was achieved for (IVb+IIa)·fcDNA. In case of duplex of tandem composed of bis-pyrene components (IVb+IIb)·fcDNA an insignificant increase of *T*_*m*_ value in comparison to (IVb+IIa)·fcDNA duplex was found. To conclude, while pyrene units at the tandem junction are likely to cause destabilization of corresponding duplexes with complementary DNA target, an attendant effect of stabilization caused by hydrophobic interactions between multiple pyrene units at the junction resulted in difference of only 2.1°C between *T*_*m*_ values of duplexes (IVb+IIb)·fcDNA and (IV+II)·fcDNA. An average *T*_*m*_ value of Series 1 tandem duplexes with fcDNA was found to be about 50°C. This confirms the formation of stable duplexes at the temperatures used for hybridization analysis.

### 3.3. Fluorescent Properties of Tandem Probes and Their Duplexes with DNA Targets

Fluorescence emission spectra of duplexes between tandems and their individual components with fcDNA target were recorded at 25°C in amplification buffer (see details in Figure 2 and in Section 2.7. at excitation wavelength of 345 nm. Amplification buffer as a medium for probes and their duplexes was chosen as we are planning to use the probes for mismatch detection directly after the amplification of a gene fragment. Preliminarily found quantum efficiencies (see Table 1) for every conjugate revealed a substantial quenching of pyrene fluorescence. Given the quantum efficiency of individual pyrene fluorophore to be 32% [30] in cyclohexane, the reduction by 25–99% was found for the studied conjugates. This finding agrees with previous reports on the quenching of pyrene fluorescence through photoinduced electron transfer to heterocyclic bases when pyrene is attached to an oligonucleotide. The extent of quenching depends on the nearest environment, that is, the nature of neighboring heterocyclic bases [44].

**Figure 3:**
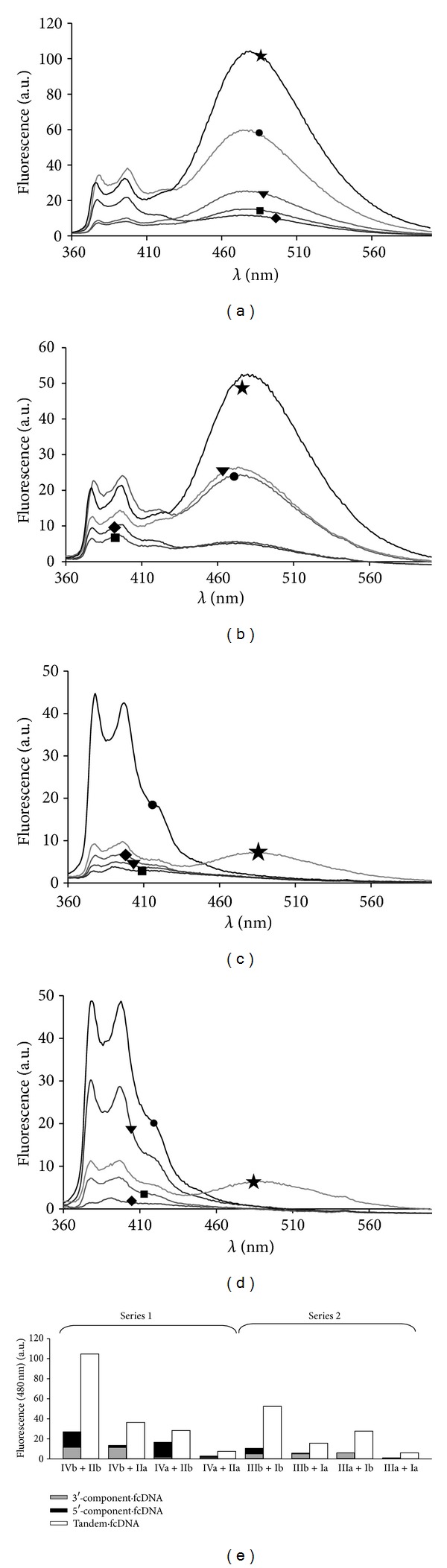
((a)–(d)) Fluorescent spectra of individual tandem components (*⚫* = 3′-component, ▾ = 5′-component), their duplexes with fcDNA (◆ = 3′-component·fcDNA, ■ = 5′-component·fcDNA), and duplexes between tandems and fcDNA (★) for the following tandems: IVb+IIb (a), IIIb+Ib (b), IVa+IIa (c), IIIa+Ia (d). (e) Fluorescence intensity values at 480 nm for duplexes of individual tandem components (gray and black) with fcDNA in comparison with that of tandem duplexes with fcDNA (white). Conditions: 6.7 mM Tris-HCl (pH 8.9), 16 mM (NH_4_)_2_SO_4_, 3.6 mM MgCl_2_, *λ*_ex_ = 345 nm, [conjugate] = [fcDNA] = 2 · 10^−7^ M, *T* = 25°C.

Upon the formation of duplexes between individual tandem components and fcDNA target we observed further quenching of pyrene fluorescence (see Figures 3(a)–3(d)). Interestingly, in the case of bis-pyrene conjugates Ib–IVb excimer fluorescence (at 480 nm) has been more significantly quenched (reduction by 1.7–5.3 times) than monomer fluorescence (at 392 nm) (reduction by 1.2–2.2 times) (see Figures 3(a) and 3(b)). For the monopyrene conjugates Ia–IVa the reduction of monomer fluorescence upon duplex formation ranged from 1.3 (for IIa·fcDNA) to 18 (for IIIa·fcDNA) times (see Figures 3(c) and 3(d)). In the presence of both tandem components a considerable increase of excimer fluorescence occurred in case of tandems composed of two or at least one bis-pyrene components (see Figures 3(a) and 3(b)). As it was expected, the largest increase of excimer fluorescence was observed for tandems possessing four pyrene units at the junction (increase by about 8 times for (IVb+IIb)·fcDNA and by about 10 times for (IIIb+Ib)·fcDNA). The stabilization of excimers is achieved if a possibility of interaction between tandem units attached to the different tandem components appears. This is confirmed by the fact that excimer fluorescence of tandem duplexes is significantly larger than the sum of excimer fluorescence intensities of duplexes of individual components (see Figure 3(e)). In case of tandems composed of monopyrene components ((IVa+IIa) and (IIIa+Ia)) an excimer signal rises in the presence of both tandem components in the duplex (see Figures 2(c) and 2(d)).

Diagram presented in Figure 2(e) does not only reflect the tendencies of fluorescent spectra changing upon hybridization of individual tandem components and tandems with fcDNA target described above, but also allows comparing two series of tandems. We found that the largest excimer fluorescence value was observed in (IVb+IIb)·fcDNA duplex (Series 1). The analogous duplex (IIIb+Ib)·fcDNA (Series 2) displays a 2 times lower value of excimer fluorescence. This can be explained either by more pronounced excimer fluorescence of individual components of tandem (IVb+IIb) or by more favorable environment of pyrene units in duplex of this tandem.

Thus we have shown an efficient excimer formation in the duplexes of tandem probes with fcDNA target. This phenomenon is the evidence of the tandem probes ability to detect specific DNA targets.

### 3.4. Application of Tandem Probes for Mismatch Detection in DNA Targets by Fluorescent Emission

Tandems composed of monopyrene conjugates of oligodeoxyribonucleotides have previously been reported to discriminate SNPs in model DNA targets by fluorescence emission [6, 8, 11]. These probes displayed smaller specific fluorescent signal upon hybridization with mismatched DNA target in comparison to fully complementary DNA target.

Herein, we applied the designed tandem probes for the systematic study of the effect of variation the number of pyrene units at the tandem junction and position of tandem junction relatively to mismatch in DNA target on their mismatch-discriminating abilities.

**Figure 4:**
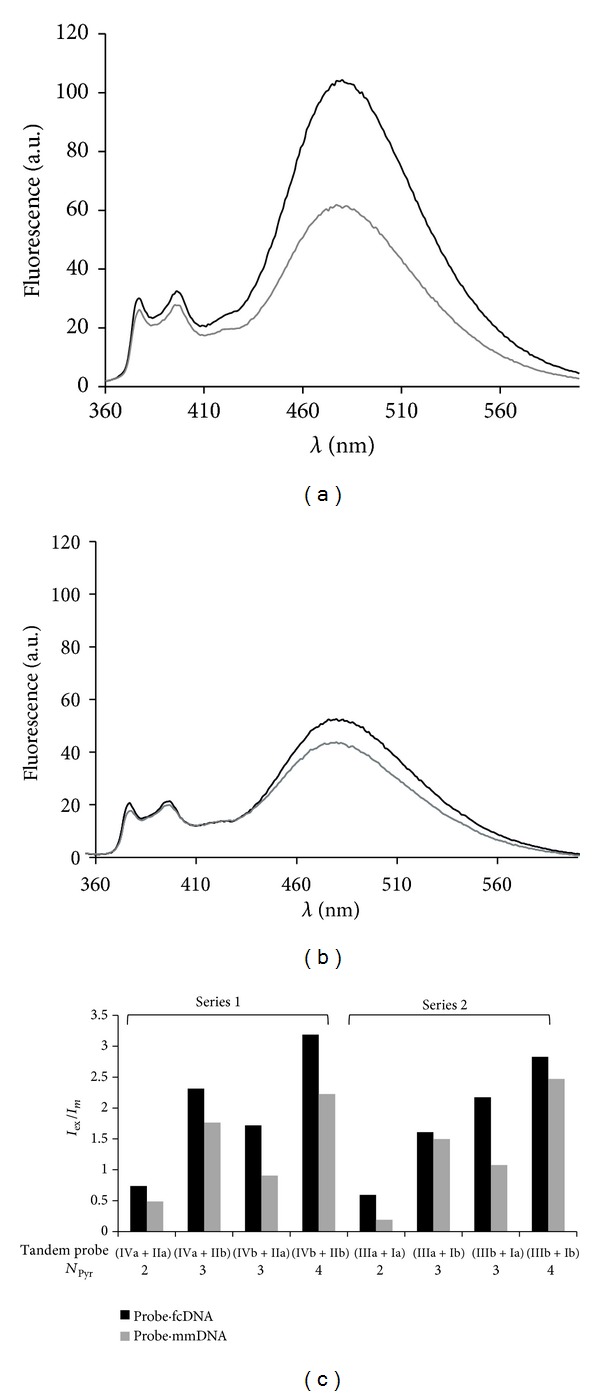
Fluorescent spectra of mismatched (probe·mmDNA) (gray) and fully complementary (probe·fcDNA) (black) duplexes of tandem probes IVb+IIb (A) and IIIb+Ib (B); C) *I*_ex_/*I*_*m*_ values for the mismatched (gray) and fully complementary (black) duplexes of tandem probes (Series 1 and Series 2). See conditions of fluorescent spectra recording under Figure 3 and in Section 2.7.

Decrease of excimer fluorescence as a result of hybridization with mmDNA in comparison to hybridization with fcDNA was typical for both tandem series (Figures 4(a) and 4(b)). To compare tandem probes by ability of mismatch-discrimination we used a ratio of monomer to excimer fluorescence (*I*_ex_/*I*_*m*_) that serves as a criterion of excimer formation [45]. We assume that the larger difference between *I*_ex_/*I*_*m*_ values for matched and mismatched duplexes is the evidence of the greater mismatch-discriminating ability of this tandem.  Figure 3(c) shows a comparative graph that reflects a relationship between mismatch-discriminating abilities of tandem probes, the number of pyrene residues at the tandem junction, and position of the junction. According to Figure 4(c) all the tandem probes were able to discriminate single nucleotide substitution in DNA targets to a certain extent. Probes of Series 1 exhibited reduction of *I*_ex_/*I*_*m*_ values from 24% (for VIa+IIb) to 47% (for VIb+IIa) upon mismatch detection. A wider range of sensitivities towards mismatch was found within Series 2. In this series we found a reduction of *I*_ex_/*I*_*m*_ values from 7% (for IIIa+Ib) to 67% (for IIIa+Ia). It is notable that for both series the least pronounced reduction of *I*_ex_/*I*_*m*_ values was typical for probe composed of 3′-mono- and 5′-bis-pyrene components. Besides, in both tandem series the probes composed of 3′-bis- and 5′-monopyrene components displayed significant sensitivity to mismatch (reduction of *I*_ex_/*I*_*m*_ values by 47% and 50% for Series 1 and Series 2 probes, correspondingly). Series 1 and Series 2 were different in the response of probes composed of two monopyrene or two bis-pyrene components to hybridization with mismatched DNA target. In case of tandems composed of monopyrene components more prominent discrimination of mismatch was found for Series 2 (tandem IIIa+Ia). For tandems of bis-pyrene components the highest mismatch-discriminating ability was found in case of Series 1 (tandem IVb+IIb). As we took into account not only the sufficient ability of tandem probes to discriminate mismatches but also high efficiency of excimer formation in their duplexes with DNA targets, the most advantageous tandem probe for detection of mismatches in DNA by fluorescent excimer emission appeared to be probe IVb+IIb (Series 1).

### 3.5. Discrimination of Mismatch in DNA by Thermal Denaturation with Excimer Fluorescence Detection Method

**Figure 5:**
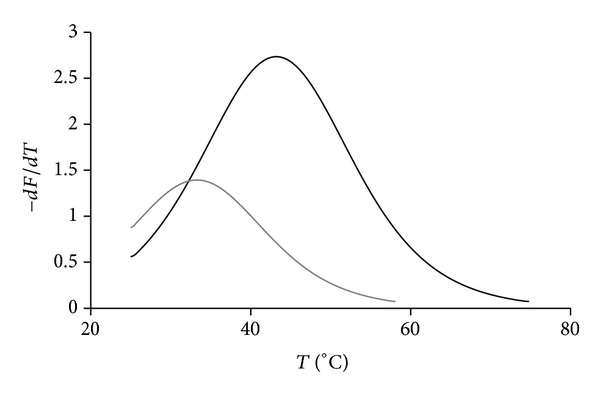
Differential melting curves of duplexes (IVb+IIb)·mmDNA (gray) and (IVb+IIb)·fcDNA (black) obtained upon excimer fluorescence detection. Melting curves were recorded with 2 · 10^−7^ M solutions of duplexes in 6.7 mM Tris-HCl (pH 8.9), 16 mM (NH_4_)_2_SO_4_, and 3.6 mM MgCl_2_. Fluorescence values were registered at 480 nm at an excitation wavelength of 345 nm upon heating of samples at a rate of 0.7–1.5°C/min.

Previously, temperature dependences of excimer fluorescence of tandem probes composed of monopyrene modified oligodeoxyribonucleotides have been studied [5, 46]. However, tandem probes have never been used before in melting curve analysis with excimer fluorescence detection for mismatch discrimination in DNA. We studied the melting curves of fully matched and mismatched duplexes of tandem probe containing two bis-pyrene components IVb+IIb registering excimer fluorescence upon heating. While heating the duplexes we observed decrease of excimer fluorescence due to melting and consequent dissociation of excimer at the junction. Since the thermal stability of mismatched duplexes is lower than thermal stability of fully matched duplexes, we were able to distinguish between them.  Figure 5 demonstrates first derivatives of melting curves for duplexes of tandem IVb+IIb with fcDNA and mmDNA targets. Difference between melting points corresponding to peaks of first derivatives of melting curves of complementary and mismatched duplexes was as considerable as 10°C.

Thus, it was possible to discriminate a single nucleotide substitution in DNA target.

### 3.6. Detection of One Nucleotide Deletions and Insertions in DNA Targets by Fluorescent Emission

**Figure 6:**
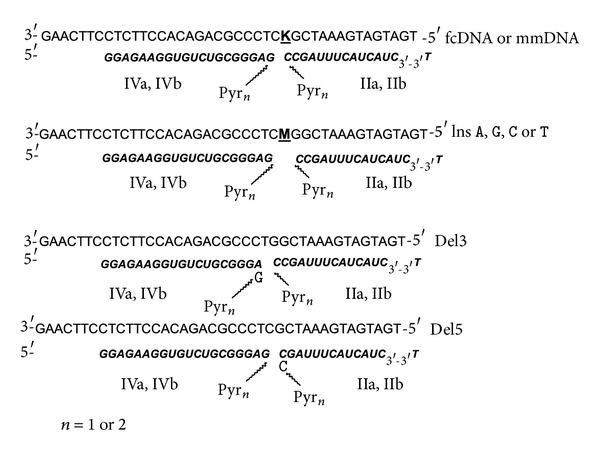
Multipyrene tandems for detection of SNP, mononucleotide insertions and deletions. N-Deoxyribonucleotide, *N*-2′-O-methylribonucleotide, **K**-G (fcDNA), or A (mmDNA), **M**-A, G, T, or C insertion; Del3 and Del5-DNA targets with mononucleotide deletion; InsA, InsG, InsC, InsG-DNA target with mononucleotide insertion (A, G, C, and G).

**Figure 7:**
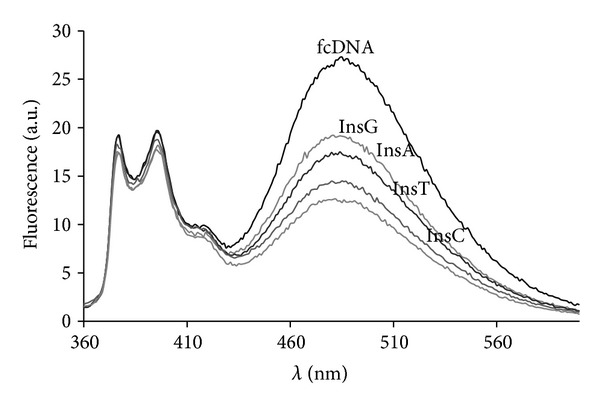
Fluorescent spectra of (IVb+IIa) duplexes with targets differing in the type of insertion (InsG, InsA, InsC, or InsT) in comparison to spectrum of duplex (IVb+IIa)·fcDNA. See conditions Figure 3.

We used the 41-mer model DNA targets containing the four variants of insertions (InsA, InsG, InsT, and InsC) to investigate the possibility of these point mutations detection with tandem probes of Series 2 (Figure 6). The insertion was supposed to increase the distance between pyrene units at the junction. We studied an effect of nature of inserted oligonucleotide (A, G, T, or C) on fluorescent spectra of duplexes between probes and corresponding targets. As it was expected, the observed extent of reduction of *I*_ex_/*I*_*m*_ values depended on the nature of the inserted nucleotide. We found more pronounced reduction for pyrimidine insertions in comparison with purine insertions. These data correlate with the previously established order of pyrene-quenching reactivities by flanking DNA bases: A<G<T<C [44]. The most prominent visible distinction between fluorescent spectra of duplexes with different insertions was found in case of tandem (IVb+IIa) (Figure 7). The reduction of excimer fluorescence followed the order G<A<T<C insertion. Comparison of the *I*_ex_/*I*_*m*_ values showed weak distinction between different insertions (Table 3). The highest reduction (~50%) of *I*_ex_/*I*_*m*_ values was typical for (IVb+IIa) and (IVa+IIa) duplexes with InsC target. A deviation from the observed change in *I*_ex_/*I*_*m*_ values was found only in case of adenosine insertion for tandems (IVa+IIa) and (IVa+IIb), when we observed the increase of *I*_ex_/*I*_*m*_ values. The reason of this phenomenon is not clear at the moment and is a subject of future studies.

**Figure 8:**
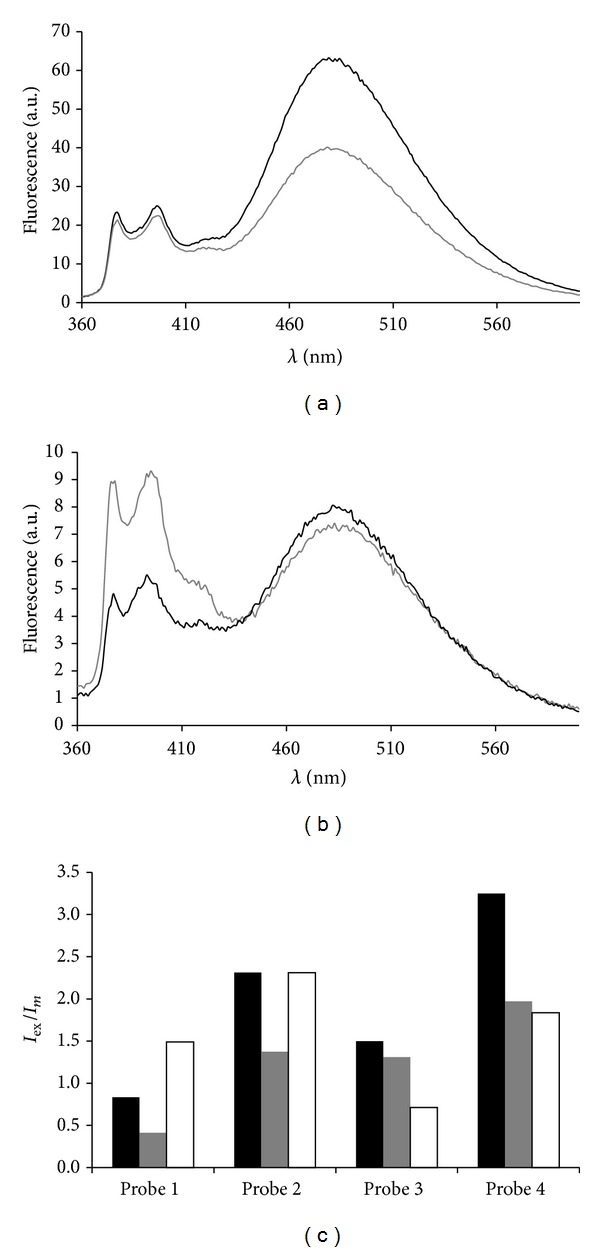
(a) Fluorescent spectra of (IVb+IIb) duplexes with fcDNA target (black) and Del3 target (gray), (b) fluorescent spectra of (IVa+IIa) duplexes with fcDNA target (black) and Del5 target (gray), (c) criterion *I*_ex_/*I*_*m*_ for duplexes of probe 1 (IVa+IIa), probe 2 (IVa+IIb), probe 3 (IVb+IIa), and probe 4 (IVb+IIb) with fully complementary fcDNA (black) and for duplexes with Del3 (gray) and Del5 (white) targets. See conditions in Figure 3.

To evaluate the influence of deletions in DNA target on fluorescent spectra of its duplexes with tandem probes we used two types of 41-mer DNA targets: Del3 and Del5 with deletion located in the first position at 3′- or 5′-side of the tandem junction, correspondingly (see Figure 6). Thus 3′- or 5′-overhang at the junction was formed upon hybridization of tandem components with DNA targets. Single nucleotide deletion may be expected to be either a factor that brings together adjoining termini of tandem components consequently leading to increase of excimer fluorescence or a factor that can disturb a favorable excimer conformation. In this study, we observed both possibilities. Fluorescent spectra of duplexes between probes and Del3 target revealed reduction of excimer formation (*I*_ex_/*I*_*m*_) for all types of probes (Figure 8(c)). While the highest reduction was found for the tandem (IVa+IIa) (~50%), the fluorescent intensity of duplexes with this probe was low (not exceeding 10 a.u.). Tandem (IVb+IIb) appeared to be the most advantageous for the detection of deletion in Del3 target as it showed a sufficient reduction of *I*_ex_/*I*_*m*_ values (~40%) along with high fluorescence intensity (~60 a.u. at the highest point). Typical fluorescent spectra of (IVb+IIb) duplexes with fcDNA and Del3 targets are shown in Figure 8(a). When studying fluorescent spectra of tandem probes duplexes with Del5 target we faced quite different changes for certain probes. Fluorescent spectra change for (IVb+IIa) and (IVb+IIb) in duplexes with Del5 target was found to be in agreement with data obtained for duplexes of these probes with Del3 target. The 3′-overhang formed in these duplexes resulted even in more prominent reduction of *I*_ex_/*I*_*m*_ values (~52 and 44% in comparison with ~13 and 39%, correspondingly). We observed ~79% increase of excimer formation for duplex of tandem probe (IVa+IIa) with Del5 target (Figure 8(b)). Remarkably, in case of (IVa+IIb) duplex with Del5 target we obtained no change in *I*_ex_/*I*_*m*_ values.

We can conclude that the designed tandem probes can be used as instruments of insertion and deletion detection in DNA target using the method of registration of excimer fluorescence change.

## 4. Conclusions

Novel tandem constructions composed of 3′- and 5′-mono- and bis-pyrene-labeled oligo(2′-O-methylribonucleotides) were proposed as instruments for point mutations detection in DNA target. A comparative research was carried out upon new tandem probes. It was shown that fluorescent properties of tandem probes and their sensitivity to mismatch, insertion, and deletion in DNA target strongly depend on the number of pyrene residues at the tandem junction and the location of mismatch position relatively to the tandem junction. The comparison and analysis of fluorescence spectra of tandem probes with DNA targets allow a facile detection of point mutation in DNA target. Analysis of melting curves of fully matched and mismatched tandem probe·DNA target duplexes obtained by detection of excimer fluorescence changing during the melting is expected to be more relevant in application to SNPs detection in amplified DNA fragments directly after PCR. On the basis of the data obtained we found that tandem probe possessing four pyrene units at the junction opposite to mismatch position displays the highest excimer fluorescence and the strongest mismatch discrimination and deletion and insertion detection ability. We have demonstrated the use of new tandem probes for the detection of C677T substitution in MTHFR gene in application to model systems. Studies involving application of the proposed tandem probes for C677T SNP detection in DNA fragments amplified by PCR are in progress.

## Figures and Tables

**Table 1:** Mono- and bis-pyrene-labeled oligo(2′-O-methylribonucleotides).

*N* ^a^	Structure	Yield^b^	Φ_*F*_	Calculated (M)^c^	Found (M)^d^
Ia	5′-**Pyrp**G^m^A^m^G^m^C^m^C^m^G^m^A^m^U^m^U^m^U^m^C^m^A^m^U^m^C^m^A^m^_3′-3′_ ***T***	67	7.6	5543.71	5542.42
IIa	5′-**Pyr**pC^m^C^m^G^m^A^m^U^m^U^m^U^m^C^m^A^m^U^m^C^m^A^m^U^m^C^m^_3′-3′_ ***T***	51	0.4	5121.42	5122.47
IIIa	5′-G^m^A^m^A^m^G^m^G^m^A^m^G^m^A^m^A^m^G^m^G^m^U^m^G^m^U^m^C^m^U^m^G^m^C^m^G^m^G^m^p**Pyr**	29	16.9	7138.77	7138.60
IVa	5′-G^m^G^m^A^m^G^m^A^m^A^m^G^m^G^m^U^m^G^m^U^m^C^m^U^m^G^m^C^m^G^m^G^m^G^m^A^m^G^m^p**Pyr**	23	16.7	7154.77	7155.50
Ib	5′**-(Pyr)**_2_pG^m^A^m^G^m^C^m^C^m^G^m^A^m^U^m^U^m^U^m^C^m^A^m^U^m^C^m^A^m^_3′-3′_ ***T***	81	4.8	5756.99	5756.23
IIb	5′-**(Pyr)**_2_pC^m^C^m^G^m^A^m^U^m^U^m^U^m^C^m^A^m^U^m^C^m^A^m^U^m^C^m^_3′-3′_ ***T***	65	14.4	5334.69	5337.87
IIIb	5′-G^m^A^m^A^m^G^m^G^m^A^m^G^m^A^m^A^m^G^m^G^m^U^m^G^m^U^m^C^m^U^m^G^m^C^m^G^m^G^m^p**(Pyr)**_2_	34	17.1	7352.05	7352.20
IVb	5′-G^m^G^m^A^m^G^m^A^m^A^m^G^m^G^m^U^m^G^m^U^m^C^m^U^m^G^m^C^m^G^m^G^m^G^m^A^m^G^m^p**(Pyr)**_2_	22	23.8	7368.05	7369.40

^a^Indexes a and b designate monopyrene and bis-pyrene derivatives of 5′- and 3′-phosphorylated oligo(2′-O-methylribonucleotides) I, II, III, and IV. ^b^Yield at the stage of pyrene coupling. ^c^Calculated molecular mass of fully protonated form. ^d^Molecular mass found by MALDI TOF MS analysis. N^m^: 2′-O-methyl-ribonucleotide; p: terminal phosphate; **Pyr**: pyrenylmethylamine residue; _3′-3′_ ***T***: 3′-“inverted” thymidine.

**Table 2:** Thermal stability^a^ of duplexes between tandem probes and their components with fcDNA target^b^.

Tandem	IVb + IIb	IVb + IIa	IVa + IIb	IVa + IIa	IVb + II	IV + IIb	IVa + II	IV + IIa	IV + II	II
*N*_pyr_^c^	4	3	3	2	2	2	1	1	0	—
*T*_*m*_, °C	51.2	50.8	49.3	49.7	45.8	49.7	53.4	48.8	53.3	42.0

^a^Conditions of thermal stability determination are listed in experimental section. ^b^For fcDNA sequence see Figure 1. ^c^Number of pyrene residues at the junction.

**Table 3:** Criterion *I*_ex_/*I*_*m*_ for fully complementary tandem duplexes and duplexes containing mononucleotide insertions.

Tandem	fcDNA	InsG	InsA	InsT	InsC
IVa + IIa	0.8	0.7	1.3	0.7	0.4
IVa + IIb	2.3	2.1	2.6	2.1	2.3
IVb + IIa	1.5	1.2	1.0	0.8	0.8
IVb + IIb	3.2	2.6	2.2	2.2	2.1
